# Low‐Cost and High‐Efficiency Solar‐Driven Vapor Generation Using a 3D Dyed Cotton Towel

**DOI:** 10.1002/gch2.201900004

**Published:** 2019-05-22

**Authors:** Yudi Yang, Yujin Sui, Zaisheng Cai, Bi Xu

**Affiliations:** ^1^ Key Laboratory of Science and Technology of Eco‐Textiles Ministry of Education College of Chemistry Chemical Engineering and Biotechnology Donghua University 2999 North Renmin Road Shanghai 201620 China

**Keywords:** dyed cotton towels, heat localization, solar‐driven evaporation, water purification

## Abstract

Solar‐driven vapor generation is a promising method to mitigate freshwater shortage and water contamination. However, most of the current highly efficient solar evaporators suffer from low robustness, tedious preparation procedures, and high cost. In this study, an easy‐to‐manufacture, low‐cost, and high‐reliability solar‐driven evaporator is designed using a black cotton towel with a hollow conical shape. The reactive dye molecules diffuse into the cotton and form strong covalent bonds with the fiber after dyeing, which firmly fixes light‐absorbing materials on the substrate. The looped pile structure of towels and hierarchical structure of yarns enable the evaporator enlarged surface area. The hollow conical shape of the cotton towel can effectively suppress the heat loss to the environment without compromising light absorption. The 3D vapor generator exhibits an evaporation rate of 1.40 and 1.27 kg m^−2^ h^−1^ for pure water and saline water, respectively. Meanwhile, this towel‐based solar‐driven evaporator exhibits a promising antifouling property as well as superior reusability and provides a reliable pathway in dealing with realistic waters, such as seawater and dyeing sewage. Therefore, the low‐cost, solar‐driven water evaporation system offers a complementary approach for high‐efficiency vapor generation and water purification in practical application.

## 1 Introduction

Solar energy, serving as the cleanest and most abundant renewable energy source, can be harnessed for a variety of applications, including power generation, absorption chillers, desalination system, and water purification.^1^, ^2^, ^3^, ^4^ Meanwhile, mankind is facing a growing global freshwater crisis. On one hand, almost 97% of all water on Earth is saline and it is largely unsuitable for direct use. On the other hand, water pollution further exacerbates the existing challenge of freshwater scarcity by contaminating excessive amounts of available water. Therefore, the world's thirst for fresh water is becoming one of the largest global risks in terms of potential impact in the 21st century.

Solar‐powered technology such as solar still offers an environmentally friendly and easily accessible solution to cope with the water crisis.^5^, ^6^ Conventional solar still exhibits low photothermal conversion efficiency due to the bulk water heating by placing the light absorber at the bottom of the water tank. Interfacial solar vapor generation, which can efficiently absorb solar light and localize the converted heat into a small amount of water at the water–air interface, enables a substantially improved efficiency of solar energy utilization. A typical bilayer structured interfacial solar vapor generator is performed by the cooperation of efficient solar light absorption, minimization of heat loss, and significant capillary action.^7^, ^8^, ^9^ The top layer acts as a solar thermal converter which can efficiently absorb the light and then convert the light to heat energy. A variety of photothermal materials, such as carbonaceous materials (carbon nanotube, carbon nanoparticle, graphene, graphite, etc.), plasmonic metals (Au, Ag, Al, etc.), semiconducting nanoparticles (black titania, Ti_2_O_3_, Fe_3_O_4_, MnFe_2_O_4_, CoFe_2_O_4_, etc.), and polymers (polypyrrole (PPy), polydopamine, etc.) have been developed for efficient solar absorbance in the past decade.^6^, ^10^ The bottom layer acts as a thermal insulator which suppresses heat loss to the underlying bulk water. Furthermore, wicking channels are essential to sustain a continuous water supply from bulk water to the evaporating surface. Despite the tremendous efforts devoted to developing various solar‐driven vapor generators,^11^, ^12^, ^13^, ^14^, ^15^, ^16^ there are still some existing limitations which greatly hinder the practical application of these devices. These limitations include i) large‐scale consumption of expensive materials such as noble metals,^11^, ^17^, ^18^ ii) inferior reliability due to the weak adhesion between the absorber and the substrate,^19^ and iii) salt accumulation in the vapor generators under continuous water evaporation.^20^ Therefore, the grand challenge we are facing now is how to effectively produce an easy‐to‐manufacture solar‐driven vapor generator with a high performance, low cost, and superior durability.

Another main challenge is how to further enhance the vapor generation efficiency. Very recently, Zhu et al.^21^ and Gan et al.,^22^ respectively, reported highly efficient solar vapor generators which show a near 100% solar‐to‐vapor conversion efficiency through careful structural design. The vapor generator will gain energy from the surrounding environment due to its lower temperature than that of the environment. In addition, solar vapor generators with a variety of geometries have been proposed to improve their solar energy or vapor conversion efficiencies, such as carbonized mushrooms,^23^ 3D artificial transpiration device,^24^, ^25^ and origami system.^26^ It is clear that improving the solar evaporation area can substantially enhance the solar‐driven vapor generation rate and photothermal conversion efficiency.

In this work, we report an inexpensive, durable, scalable, and efficient solar‐driven vapor generator composed of a 3D dyed black cotton towel and an insulating polystyrene (PS) foam. Cotton towel is one of the most accessible and abundant resources with low cost. The unique looped pile structure and the macro‐view conical shape of our prepared dyed towel (DT) in this study endow itself efficient light absorption and low light reflection. Meanwhile, the solar‐driven vapor generator exhibits superior durability due to the strong covalent bonds between the reactive dye molecules and cotton fibers by simple dyeing technology. Our solar vapor generator exhibits an evaporation rate of 1.40 kg m^−2^ h^−1^ under one‐sun illumination with reliable cyclicity. Meanwhile, our device shows an ideal vapor generation and water purification performance for both simulated seawater and dyeing sewage. This durable, cost‐effective, scalable, and efficient solar‐driven vapor generator provides the potential to mitigate freshwater shortage and water pollution through solar energy.

## 2 Results and Discussion

### 2.1 Preparation and Characterization of Dyed Cotton Towel

**Figure 1:**
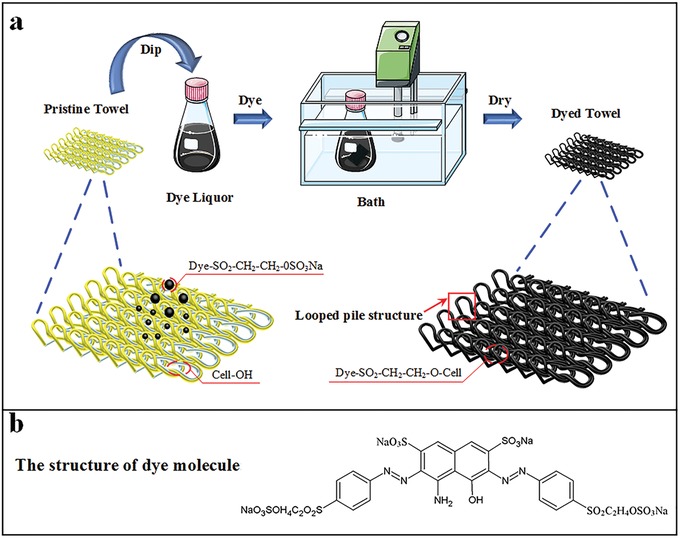
a) Schematic diagram of the dyeing process. b) Chemical structure of the main reactive dye molecule.

In this work, a black cotton towel was employed as the solar light absorber for several reasons: i) low cost, ii) high water absorbency, iii) good mechanical durability, and iv) flexibility. Moreover, textile dyeing has been developed for thousands of years and regarded as a mature technology. As shown in **Figure** **1**, the cotton towel was submerged in a dyeing solution containing dyes, salt (Na_2_SO_4_), and alkali (Na_2_CO_4_ and NaOH). Dye molecules are fixed to the fiber by absorption, diffusion, and bonding with temperature and time being key controlling factors. The commercial reactive dye was chosen due to its strong bonding between dye molecules and cotton fiber. The dye molecules possessing reactive groups were attached to the fiber by covalent bonding to form “dye‐fiber” compound (Figure S1, Supporting Information). Therefore, the reactive dye shows good color fastness properties to washing (grade 4–5), rubbing (grade 4–5), and light (grade 3–4) owing to the covalent bonding that occurs during dyeing (Table S1, Supporting Information).^27^ Comparing with most of the previous solar absorbers which were prepared by the deposition of photothermal materials onto the supporting substrates via vacuum filtration,^17^, ^19^ this dyed cotton towel shows superior durability.

**Figure 2:**
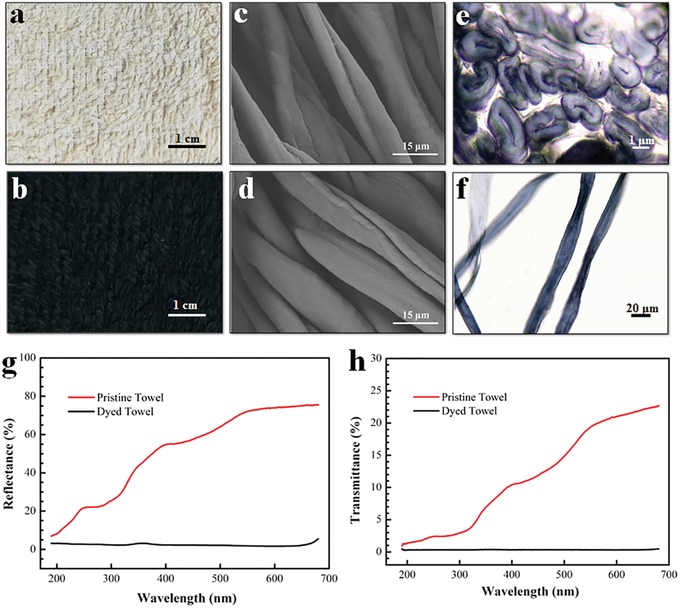
The digital images of a pristine towel a) before and b) after dyeing. SEM images of the cotton fiber c) before and d) after dyeing. e) Cross‐sectional and f) longitudinal microscope images of the dyed fibers. g) Transmittance and h) reflectance spectra of the dyed cotton towel.

The pristine towel (**Figure** **2**a) turned black after dyeing (Figure 2b), an intuitive indication of the efficient light absorption. A cross‐sectional microscope image of the dyed fibers (Figure 2e) demonstrates that lumens of these cotton fibers became dark, indicating a deep penetration of the dye molecules into the fibers. Meanwhile, a longitudinal view of the dyed cotton fibers (Figure 2f) exhibits a complete coverage of the dyestuff on fibers. Scanning electron microscope (SEM) images display no distinct variations on the surface morphology of fibers before (Figure 2c) and after dyeing (Figure 2d). This is because the reactive dye is a kind of small molecular dye which can infiltrate into the inner space of cotton fibers. In comparison with the other technologies which deposit the absorbing materials on the outer surface of supporting materials, the converted solar energy via the solar absorbers (dye molecules) can be localized in the fiber skeleton and utilized in situ to heat the small amount of water confined in the cellulose fibers, further decreasing the convective heat loss and improving the solar photothermal conversion efficiency. The optical absorption of the cotton towels before and after dyeing was recorded by a UV‐vis–NIR (near‐infrared) spectrophotometer equipped with an integrating sphere. In comparison with the pristine towel, the dyed cotton towel shows a negligible transmittance (≈0%) and a quite low reflectance (≈2.5%) in the ultraviolent and visible range (Figure 2g,h). The dyed black cotton towel has an absorbance of about 95% over a wavelength range from 250 to 680 nm, enabling efficient solar energy harvesting. It is believed that the optical absorbing property of the black dye is the main contributor to the high light absorption of the dyed cotton towel. Meanwhile, the typical looped pile structure and extremely porous structure of cotton towel further increase the optical path and then decrease the reflection, which also makes a contribution in this aspect (Figure S2, Supporting Information).

### 2.2 Wetting Property of Cotton Towels

**Figure 3:**
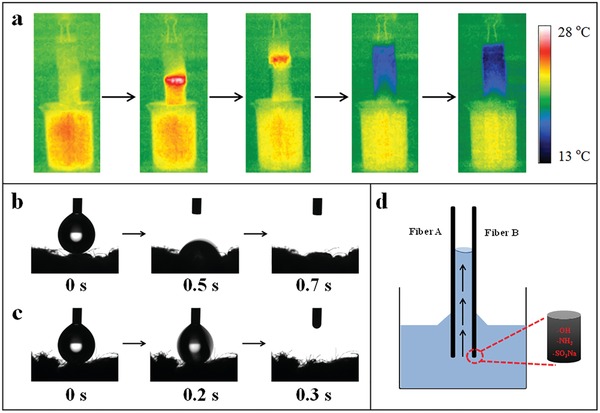
a) Infrared images of the wetting process of a cotton towel strip. Pictures of the wetting process of the cotton towel b) before and c) after dyeing. d) Schematic of wicking of the dyed cotton fibers.

The wicking property of photothermal materials is crucial to the performance of a solar vapor generator. Efficient and adequate water supply to the water–air interface is essential to achieve heat localization and high evaporation rates. The wicking ability of the dyed cotton towel was evaluated by monitoring its wetting process once in touch with water using a portable infrared camera. A fabric strip (6 cm × 2 cm) was suspended vertically with its bottom end dipped into a reservoir of water. It is interesting that the temperature of the cotton towel increased slightly at the initial stage and then decreased (**Figure** **3**a). The immediate temperature increase of the cotton towel after being dipped into water is because the towel absorbs water and releases absorption heat simultaneously while the evaporation heat is zero at first. As the water evaporates and water vapor diffuses from the fabric surface into the surrounding air, the surface temperature of the wetted cotton towel decreases. These demonstrate that our cotton towel shows a strong wicking ability.^28^ In addition, the wettability of the pristine towel and the DT was also evaluated with a goniometer. When a water droplet (5 µL) was placed on the cotton towel, it would cost 0.7 s to infiltrate into the pristine towel (Figure 3b) while only 0.3 s was needed for the DT to complete this process (Figure 3c). It is believed that the cotton fibers colored with reactive dye possess more hydrophilic groups (i.e., amidogen, sulfonic groups, and hydroxyl groups) than the pristine cotton fibers (i.e., hydroxyl groups) (Figure 3d). These indicate cotton towels are hydrophilic while the DT displays superior hydrophilicity, which will facilitate the water transport to the evaporation surface and benefit the achievement of efficient solar vapor generation.

### 2.3 Photothermal Property of Dyed Cotton Towel

**Figure 4:**
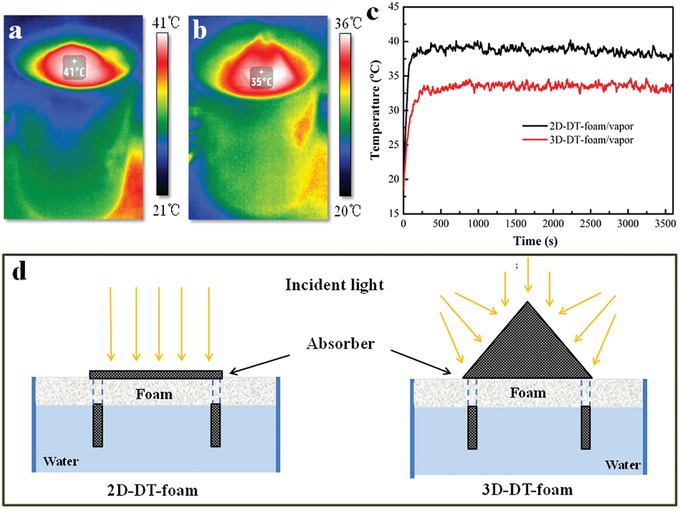
Infrared images of a) 2D‐DT‐foam and b) 3D‐DT‐foam. c) Temperature of generated vapors. d) Schematic illustration of the light absorption in different systems.

Recently, geometry‐oriented 3D structures for highly efficient solar distiller have been reported and the benefits of these structures can be generally summarized as follows: i) reducing convection and radiation losses^24^; ii) increasing the evaporation area; and iii) enhancing the solar absorption.^26^ Here, a cotton towel with a hollow conical shape was prepared by means of an assembly of the flat fabric as schematically shown in Figure S3 in the Supporting Information. Two cotton strips connecting with the conical towel was immersed in the water for sustaining water supply as well as minimizing the heat loss. An evaporator was achieved by placing the 3D DT onto a floating PS foam (3D‐DT‐foam). The photothermal property of this 3D vapor generator was investigated in comparison with its counterpart: a planar system (2D‐DT‐foam). With the initial ambient temperature being constant at ≈18 °C, the surface temperature of the 2D‐DT‐foam drastically increased to 41 °C after 15 min under one‐sun illumination (**Figure** **4**a). For comparison, 3D‐DT‐foam exhibited a lower surface temperature of 35 °C (Figure 4b) under the same condition. The less temperature increase for the 3D‐DT‐foam is attributed to its increased evaporation area and the cooling effect of consequently enhanced water evaporation. Meanwhile, the lower surface temperature of the solar absorber will effectively reduce convection and radiation losses to the environmental air, which is beneficial for the improvement of the photothermal conversion efficiency. Furthermore, a thermal couple sensor probe was employed to measure the temperature of the generated vapor. As shown in Figure 4c, the vapor temperature of 3D‐DT‐foam and 2D‐DT‐foam drastically increased to 33 and 38 °C, respectively, within 250 s and then reached a steady state in the subsequent time period. This trend is in accordance with the black cotton towel. Moreover, the conical dyed cotton towel receives solar light from all directions while the planar DT absorbs single incident light most in the vertical direction (Figure 4d). This is important for practical application since the sunlight incident angle varies during solar motion.^26^ In addition, multiple light scattering due to the unique loop structure of the towel further enhances the light trapping capability in the gaps of the fibers (Figure S2, Supporting Information), leading to improved utilization of solar energy.

### 2.4 Solar Vapor Generation Performance

**Figure 5:**
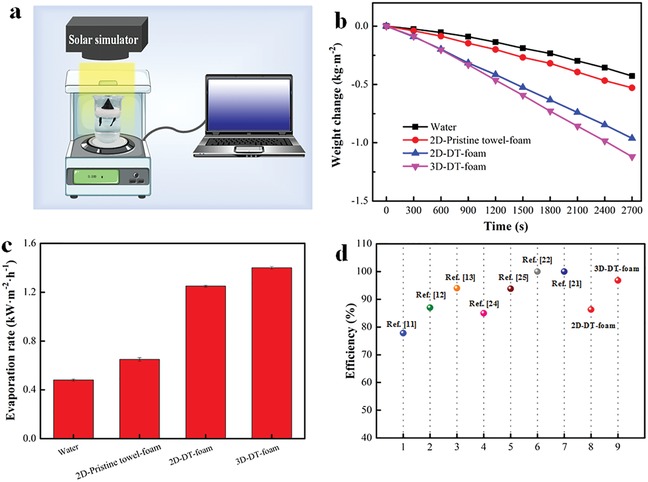
a) Schematic of the lab‐made setup for vapor generation performance measurement. b) Water mass loss curves and c) corresponding evaporation rates of different samples as vapor evaporator. d) Comparison of thermal conversion efficiency with previous work.

To evaluate the performance of the prepared solar evaporators, the evaporation rates were experimentally measured by continuously recording the mass change of water over irradiation time under one‐sun illumination and dark unilluminated conditions (**Figure** **5**a). These tests were performed at an ambient temperature of 24 ± 0.5 °C and a relative humidity of 55 ± 5%. Furthermore, all the measured evaporation weight change has already subtracted the value in the dark environment to eliminate the effect of spontaneous water evaporation (Figure S4, Supporting Information). The weight loss curves of the prepared samples display linear increase with the illumination time after an initial slow increase (Figure 5b). The evaporation rates of 2D‐DT‐foam and 3D‐DT‐foam are calculated to be 1.25 and 1.40 kg m^−2^ h^−1^, respectively (Figure 5c). Recently, a solar vapor generator with a record high evaporation rate of 3.2 Kg m^−2^ h^−1^ was reported under one sun via a hierarchically nanostructured gel based on polyvinyl alcohol (PVA) and PPy. It has been claimed that this extremely high evaporation rate is achieved through sequential cooperation of energy confinement, reduced vaporization enthalpy, and precision water management. On one hand, the converted energy via solar absorbers (PPy) can be effectively confined in the PVA network due to the insertion of PPy in the polymeric PVA gel. Therefore, the confined energy can be directly delivered to the small amount of water contained in the molecular meshes of PVA network and utilized in situ to power water evaporation. On the other hand, the water confined in the molecular mesh is proved to exhibit a reduced vaporization enthalpy comparing with that of bulk water because the confined water molecules in the PVA molecular mesh are more likely to escape the network as small clusters rather than individual molecules. Similarly, the converted solar energy in our work will directly heat the confined water in the cellulose fibers due to the penetration of dye molecules in the cotton fibers, contributing to the evaporation rate. Furthermore, some of the water clusters confined by the cellulose fiber are activated during vaporization and tend to evaporate more easily from the cotton fibers into the air, reducing the overall energy consumption and further contributing to the evaporation rate.^13^ The corresponding solar thermal conversion efficiency of 2D‐DT‐foam and 3D‐DT‐foam are calculated to be 86.2% and 96.8%, respectively. The 3D‐DT‐foam system exhibits a much higher solar thermal conversion efficiency and is among the best in comparison with the previously reported solar‐driven vapor generators (Figure 5d). However, the extremely high solar thermal conversion efficiency of our 3D‐DT‐foam evaporator seems a contravention of the principle of conservation of energy because the calculated radiation, conduction, and convection loss are 5.7%, 5.5%, and 3.0%, respectively (details are shown in the Supporting Information). Therefore, the conversion efficiency should be lower than 85.8%. This issue has been discussed in our previous work.^29^ We think the calculated conversion efficiency here is overestimated because of the much higher actual surface area than the projected area. The actual surface area of our 3D‐DT‐foam evaporator is much higher due to its conical shape, looped pile structure, and hierarchical structure of the yarn. The projected area which is utilized for calculation of vapor conversion efficiency is much lower than the actual surface area, resulting in high conversion efficiency.

**Figure 6:**
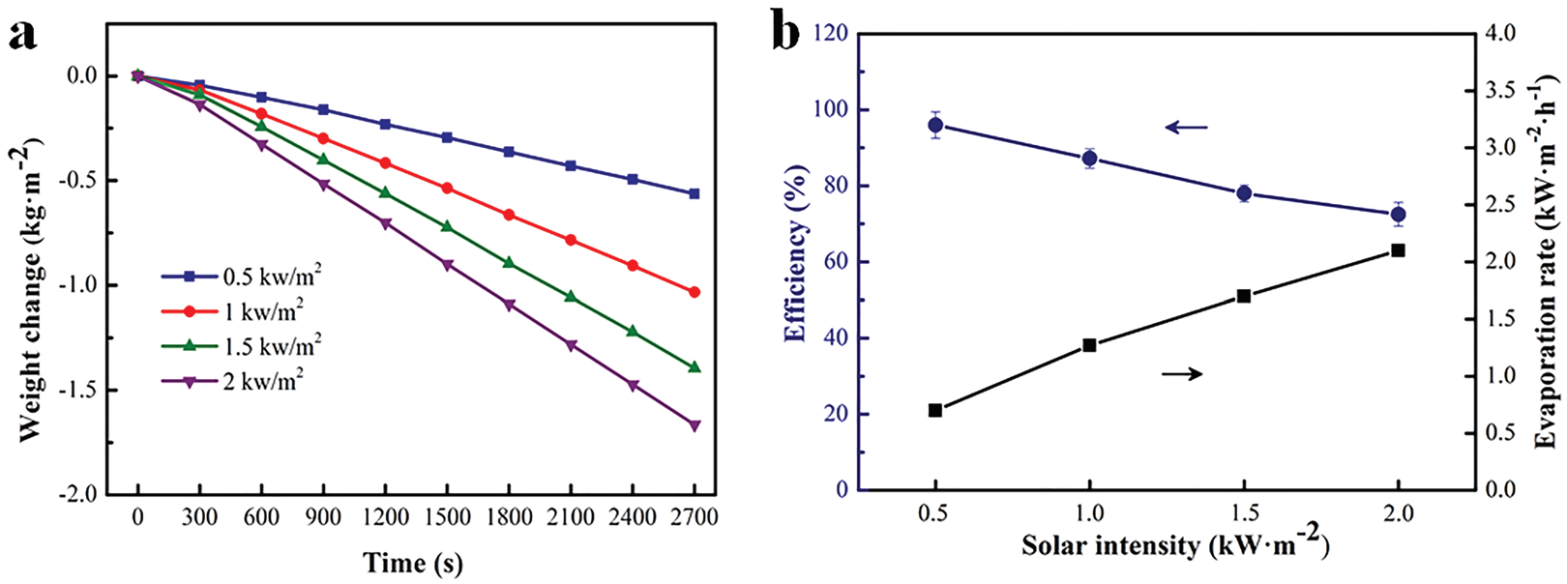
a) Solar‐driven water evaporation curves of time‐dependent weight loss under different illumination intensities. b) The solar thermal conversion efficiency and evaporation rate of 3D‐DT‐foam under different illuminations.

Besides the fresh water, a brine with 3.5 wt% NaCl was also utilized for the solar‐driven water evaporation experiment to explore the universality of our prepared sample. Here, 3D‐DT‐foam was selected to evaporate the salt water as a result of its better performance in comparison to 2D‐DT‐foam (Figure S5, Supporting Information). **Figure** **6**a shows the solar‐driven water evaporation curves of time‐dependent weight loss under different illumination intensities. The evaporation rates under the light intensities of 0.5, 1, 1.5, and 2 kW m^−2^ are 0.70, 1.27, 1.70, and 2.10 kg m^−2^ h^−1^, respectively. The corresponding solar thermal conversion efficiencies are 96.0%, 87.2%, 78.0%, and 72.5%, respectively (Figure 6b). There is a small decrease in evaporation rate and thermal conversion efficiency for the salt water compared with the fresh water. It is thought that the gradually accumulated salt particles on the surface of the photothermal fabric under continues water evaporation might deteriorate optical and wicking properties of the absorber and result in a lower evaporation rate.^30^ Although the weight loss clearly increased from 0.77 kg m^−2^ at 0.5 kW m^−2^ to 2.25 kg m^−2^ at 2 kW m^−2^ over 2700 s (Figure 6a), the solar thermal conversion efficiency slightly decreases with increasing illumination intensity. Concentrated illumination will lead to a much higher surface temperature of the absorber, leading to more heat loss from the absorber to the environment via heat exchange.^21^, ^22^ Furthermore, Zhao and Wu reported that the speed of water vapor that escapes from the absorber cannot match with the increasing absorbed solar energy. Therefore, the increased vapor pressure on the absorber would hinder the water vapor escaping from the evaporation system. In addition, the hydrophilia and looped pile structure of the cotton towel can hold water molecules in some degree.^31^ These several reasons may lead to the decreased solar thermal conversion efficiency when the solar power density increases.

### 2.5 The Reusability and Antifouling of 3D‐DT‐foam

**Figure 7:**
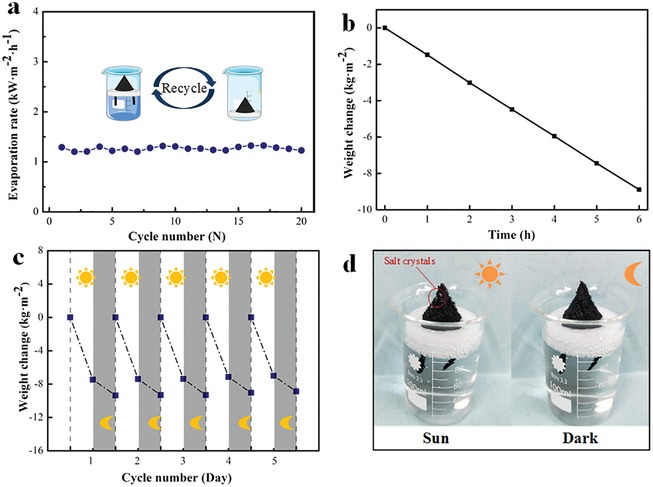
a) Reusability of 3D‐DT‐foam under one‐sun irradiation for 20 cycles with a 3.5 wt% NaCl solution. b) Mass loss of the 3D‐DT‐foam system under one‐sun illumination as a function of time within 6 h. c) Reusability of 3D‐DT‐foam with 3.5 wt% NaCl water for 5 h under one‐sun irradiation and subsequent 19 h in the dark each day. d) Salt particles accumulate under 5 h irradiation and disappear in the dark after 19 h.

Besides the evaporation rate and solar thermal convention efficiency, the reliability and antifouling property are also very important for a solar still, specifically for the application in saltwater vaporization. To demonstrate the reusability of our 3D‐DT‐foam, the solar water evaporation experiments were repeated by using saline water with 3.5 wt% NaCl. In the beginning, a 3D‐DT‐foam device was illuminated under 1 kW m^−2^ for 1 h and then dried completely in an oven to reuse for the next cycle. As shown in **Figure** **7**a, the evaporation rate is stable (i.e., 1.20–1.30 kg m^−2^ h^−1^) after 20 cycles, confirming the reliability of the proposed 3D‐DT‐foam. It can be observed from Figure 7b that the water evaporation rate of the 3D‐DT‐foam system is almost independent on the illumination time in a long time of 6 h, further demonstrating the stability of our prepared evaporator. However, salt particles can be clearly observed and gradually accumulated on the surface of the towel after long‐time continuous evaporation under illumination. This should be avoided in the practical application. Fortunately, there is a day and night cycle on Earth, which indicates two situations in one day—illumination and dark.^30^ To simulate the actual condition, the 3D‐DT‐foam evaporator was first exposed under the sunlight (1 kW m^−2^) for 5 h and then allowed to cool by putting the sample in dark for 19 h. At the start of each day, the concentration of the NaCl solution was set constant. As shown in Figure 7c, the cumulative weight loss of 3D‐DT‐foam slightly decreases from 7.50 to 7.00 kg m^−2^ for 5 h illumination after 5 days, which reveals the good reusability of our 3D‐DT‐foam. Salt crystals were observed on the surface of the fabric after 5 h illumination. However, these salt crystals disappeared at the end of one day (Figure 7d). This demonstrates that our 3D‐DT‐foam evaporator can achieve excess salt rejection and efficient vapor generation simultaneously. This is because salt rejection can occur via diffusion and advection down the fabric wick.^30^ The enhanced evaporation in the daytime takes away more water and increases local salinity. However, the fabric pumps relatively dilute seawater to the absorber with negligible evaporation in the dark and the salt particles would dissolve into the water again.

### 2.6 Application for Actual Seawater and Dyeing Wastewater

**Figure 8:**
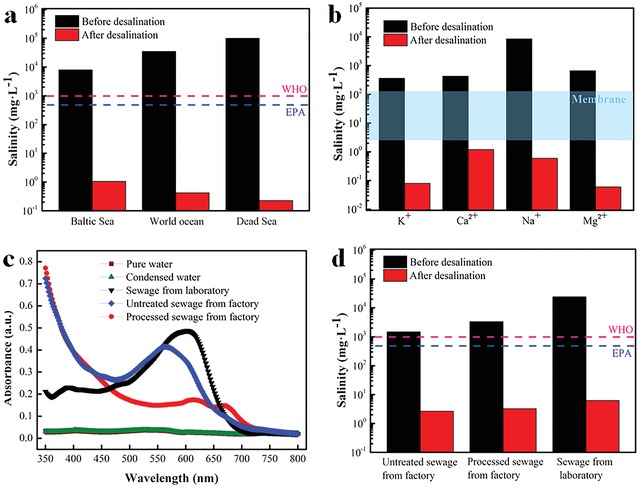
a) Measured salinities of three simulated seawater before and after desalination. b) Measured concentrations of four primary ions in an actual seawater sample before and after desalination (the blue shaded area refers to the overall typical salinity achieved by the traditional membrane desalination process). c) UV‐vis spectra of different samples. d) Measured salinities of three water samples before and after desalination.

To further evaluate the performance of our 3D‐DT‐foam system in dealing with other waters, three simulated seawater samples of different salinities—Baltic sea (lowest salinity, 0.8 wt%), World ocean (average salinity, 3.5 wt%), and the Dead sea (highest salinity, 10.0 wt%)—were prepared and tracked by an inductively coupled plasma‐optical emission spectrometer (ICP‐OES). **Figure** **8**a shows Na^+^ concentration of the condensed water significantly decreases below the salinity level defined by World Health Organization and the standard of US Environmental Protection Agency. Meanwhile, a real seawater sample (Yellow sea, China) was also utilized for test (Figure 8b). Noticeably, the concentration of four primary ions originally presenting in the seawater, namely, K^+^, Ca^2+^, Na^+^, Mg^2+^, drastically reduces to a lower level by our 3D‐DT‐foam compared with conventional membrane‐based (10–500 ppm) distillation method. In addition, to deal with the dyeing wastewater pollution, three raw sewage samples (i.e., sewage from our laboratory, untreated sewage from the factory, and treated sewage from the factory) were used to assess the purification ability of our prepared device. After sewage evaporation, the condensed water became transparent and colorless (Figure S6, Supporting Information). To further demonstrate the concentration of dye in the condensed water, the optical absorption spectra of the water samples were recorded (Figure 8c). In comparison with the sewages, the condensed water presents a negligible absorbance (i.e., near zero) at visible range (from 350 to 800 nm), which is very close to pure water. Furthermore, a mass amount of salt must be consumed for the dyeing process of the cellulose fibers, such as cotton, viscose, jute. This will produce lots of salt‐containing wastewater. However, Figure 8d shows that the salinity of the generated condensed water using our 3D‐DT‐foam evaporator is at a safety level.

## 3 Conclusion

In summary, a simple, low‐cost, and high‐efficiency solar‐driven evaporator has been achieved by a 3D dyed black cotton towel. The unique looped pile structure and the conical shape of the black cotton towel enable the device to exhibit an evaporation rate of 1.40 kg m^−2^ h^−1^ and a corresponding solar thermal conversion efficiency of 96.7% under one‐sun irradiation. In addition, the evaporator presents remarkable desalination or purification performance for seawater and dyeing sewage as well as significant reusability. Our 3D towel‐based evaporator offers a promising pathway for high‐efficiency seawater treatment and dyeing sewage purification in a large scale. In the future, how to more scientifically evaluate the evaporation performance still deserves more attention and discussion. Structure change of organic dyes may occur after long‐term solar irradiation, leading to the decreasing of solar light absorption. Therefore, coating the dyed fibers with carbon particles can further enhance the light absorbance and improve the water evaporation rates.

## 4 Experimental Section

*Reagents and Materials*: Commercial compound black reactive dye was obtained from Kyungin Synthetic Corporation (Shanghai, China). Sodium sulfate (Na_2_SO_4_), sodium hydroxide (NaOH), sodium carbonate (Na_2_CO_3_), and sodium chloride (NaCl) were provided by Sinopharm Chemical Reagent Co., Ltd. (Shanghai, China). Commercial soaping agent (ERIOPON R) was purchased from Greenexplore Textile & Chemical Co., Ltd. (Hangzhou, China). All reagents were used without further purification. Cotton towels were purchased from Shandong Kingshore Co., Ltd.

*Preparation of DT*: The dyeing process was carried out by using a water bath shaker (DL‐2003, Daelim Starlet, Korea) according to the dyeing diagram shown in Figure S7 in the Supporting Information. The cotton towel was dyed with a compound black reactive dye at the concentration of 8.88% on the weight of the cotton fabric. The bath ratio (the ratio of fabric weight and dye liquor) was kept at 1:20. First, Na_2_SO_4_ (100 g L^−1^) was added as a dyeing accelerant into the dyeing solution. The towel was then immersed in the dyeing solutions at 60 °C. After 30 min, Na_2_CO_3_ (5 g L^−1^) and NaOH (1.2 g L^−1^) which act as the fixing agents were added into the dyeing solution. To remove the residual dyes on the cotton fibers, the DT was taken out from the dyeing solution and rinsed thoroughly in distilled water and subsequently washed in hot soap solution at 90 °C for 10 min. Finally, the DT was thoroughly washed with distilled water and dried in the oven.

*Characterizations*: The morphology of the DT was characterized by SEM (TM‐1000, Hitachi, Japan) and optical microscope (MN‐59XC, Soif, China). The washing, rubbing, and light fastnesses of the DT were tested according to GB/T3921.3—2008, GB/T3920—2008, and GB/T8427—2008, respectively. The optical transmittance and reflectance spectra of the DT were measured by a UV‐vis–NIR spectrometer equipped with an integrating sphere (UV 3600, Shimadzu, Japan). The absorbance was calculated by Equation (1)

(1) A = 1−R−T

where *R* and *T* are the reflection and transmission, respectively. The surface temperature of the sample was detected by an infrared camera (SEEK Compact Pro, Seek Thermal, America). Surface wettability of the samples was measured using a goniometer (DSA30, KRÜSS, Germany). The weight change of water during evaporation was measured using an electronic mass balance with an accuracy of 1 × 10^−4^ g (ME204E, Mettler Toledo, America). The vapor generation experiments were conducted in the lab using a xenon lamp as a solar simulator (PLS‐SXE300/300UV, Perfect Light, China). The light intensity was measured by an irradiatometer (FZ‐A, Photoelectric Instrument Factory of Beijing Normal University, China). The temperature of generated vapor and bulk water was measured by a thermal couple sensor probe (DT‐8891E, CEM, China). UV‐vis spectra were utilized to determine the concentration of sewage in the condensed water (U‐3310, HITACHI, Japan). The ion concentrations of condensed water were measured by the ICP‐OES (LEEMAN LABS, America).

*Solar Vapor Generation Measurement*: A piece of PS foam with a diameter of 5 cm and a thickness of 1 cm, was placed on the water surface of a beaker (the diameter is 5.2 cm) containing 100 mL water. Both samples of 2D shape (the diameter is 2 cm) and 3D shape (the diameter is 2 cm) with apex angel of 53^o^ were placed on the foam. The evaporators were irradiated by a solar simulator under different optical concentrations. The real‐time mass change was measured by an electronic mass balance which connected with a laptop for the evaluation of the evaporation rate at a steady‐state condition and solar‐thermal conversion efficiency. Herein, Equation (2) is employed for calculating solar thermal conversion efficiency (η_th_)

(2) $$\eta_{\mathrm{th}}=\frac{\dot{m}h_{\mathrm{LV}}}{C_{\mathrm{opt}}P_{0}}$$

where $\dot{m}$ is the mass flux, *C*_opt_ *P*_0_ is the solar illumination energy (i.e., *C*_opt_ is the optical concentration and *P*_0_ is the normal direct solar irradiation), *h*_LV_ is the total enthalpy of liquid–vapor phase change containing two parts, i.e., the sensible heat (i.e., *C* × (*T* −*T*_0_)) and the enthalpy of vaporization (Δ*h*_vap_).^32^ *C* is the specific heat capacity of water and is a constant (i.e., 4.18 J g^−1^ K^−1^). *T* is the vapor temperature and *T*_0_ is the initial temperature of water, both of which were measured by a thermal couple sensor probe.

## Conflict of Interest

The authors declare no conflict of interest.

## Supporting information

Supplementary
