# Supplementary material for: Low‐Cost and High‐Efficiency Solar‐Driven Vapor Generation Using a 3D Dyed Cotton Towel
Source: Glob Chall. 2019 May 22;3(9):1900004. doi: 10.1002/gch2.201900004 (PMC6733398; doi:10.1002/gch2.201900004)
Supplement: Supplementary file 1 — Supplementary [file GCH2-3-1900004-s001.pdf]

Copyright WILEY-VCH Verlag GmbH & Co. KGaA, 69469 Weinheim, Germany, 2019.

Global Challenges

Open Access

Supporting Information

for *Global Challenges,* DOI: 10.1002/gch2.201900004

Low-Cost and High-Efficiency Solar-Driven Vapor Generation Using a 3D Dyed Cotton Towel

*Yudi Yang, Yujin Sui, Zaisheng Cai, and Bi Xu**

Copyright WILEY-VCH Verlag GmbH & Co. KGaA, 69469 Weinheim, Germany, 2019.

Supporting Information

Low-cost and High-efficiency Solar-driven Vapor Generation Using a Three-dimensional Dyed Cotton Towel

*Yudi Yang, Yujin Sui, Zaisheng Cai, and Bi Xu**

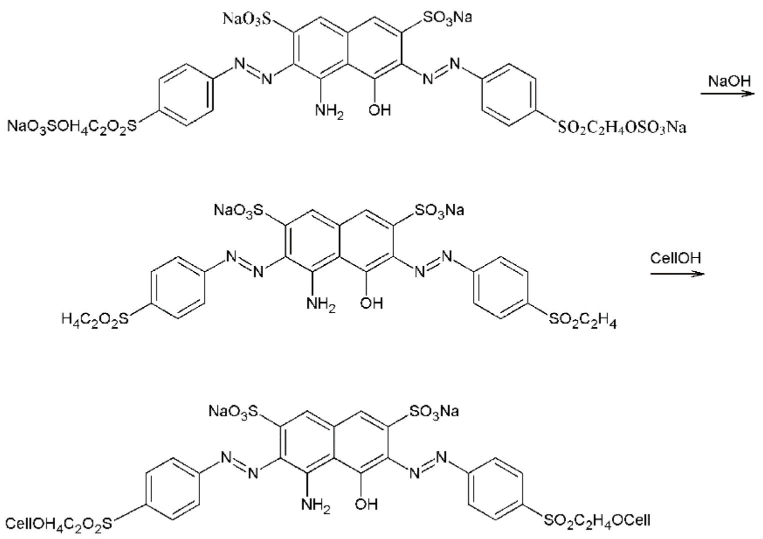

Figure S1. The reaction of the reactive dye with cotton fibers.

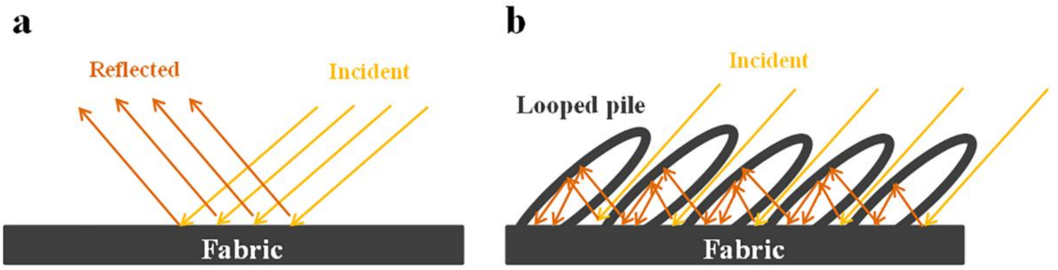

Figure S2. Schematic illustration of (a) light is reflected away from relatively smooth fabric and (b) light going through multiple internal reflections due to the looped pile structure.

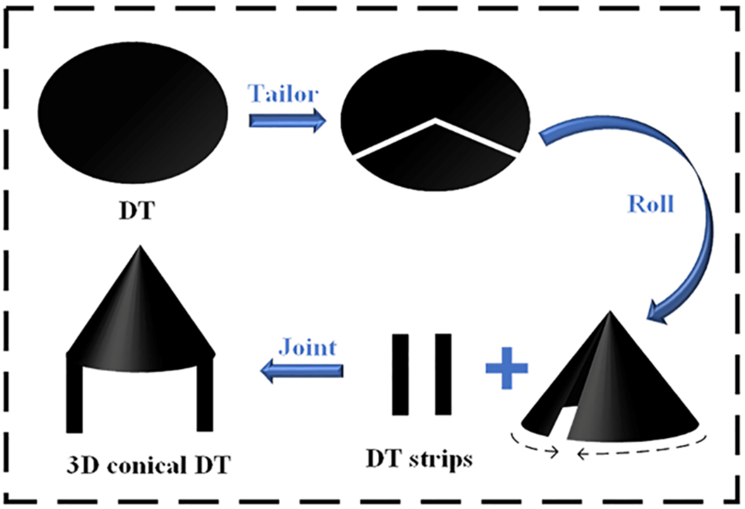

Figure S3. Schematic of the fabrication process of three-dimensional evaporator based on a conical dyed towel.

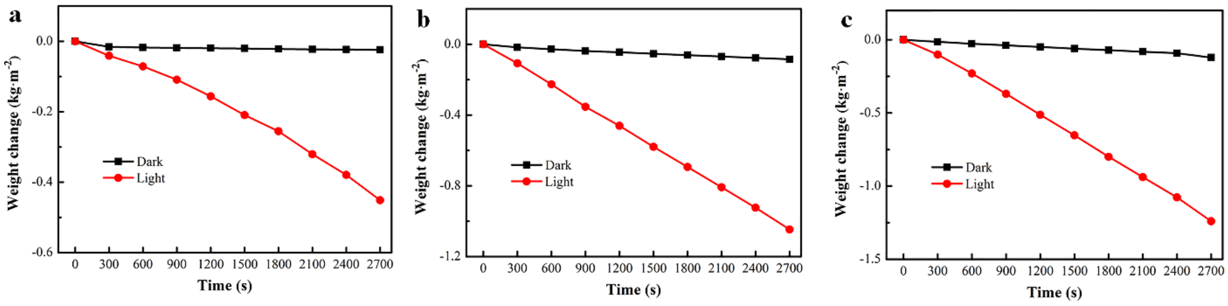

Figure S4. The weight changes of (a) pure water, (b) 2D-DT-foam and (c) 3D-DT-foam systems as a function of time with (red line) and without (black line) illumination.

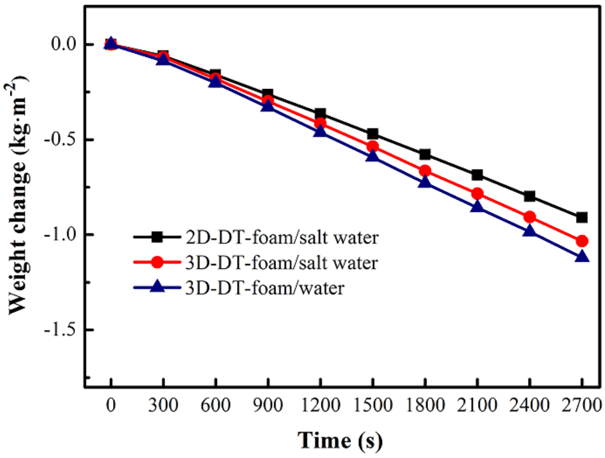

Figure S5. Weight change of different solar vapor generation systems as a function of time under 1 kW m^{-2} solar irradiation.

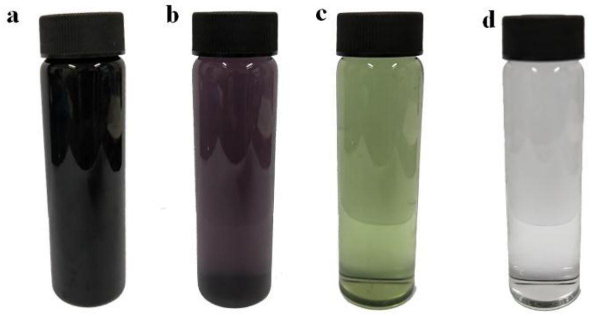

Figure S6. Photographs of (a) sewage from the laboratory, (b)untreated sewage from the factory, (c) processed sewage from the factory and (d) the collected condensed water.

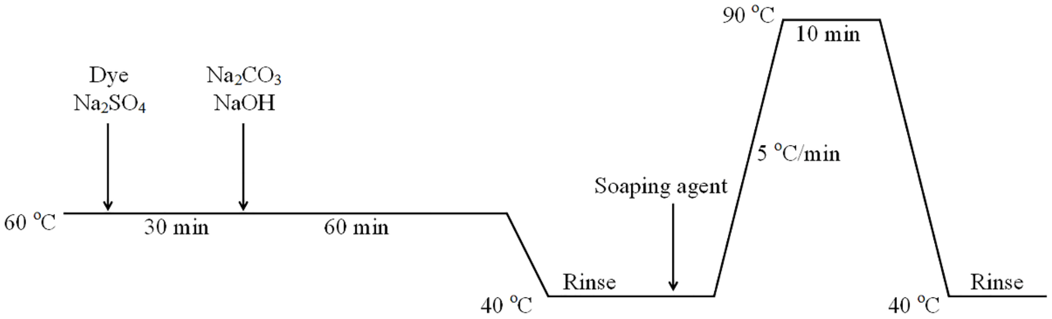

Figure S7. Dyeing profile of cotton towel with reactive dyes.

Table S1. Fastness of the dyed cotton towel.

Fastness	Wash	Rubbing		Light
		wet	dry	
Dyed towel	4-5	3-4	4-5	3-4

[(Units 1, 2, 3, 4, 5): 0 is the lowest and 5 is the highest]

Heat loss analysis

(a) Radiation loss

The radiation loss was calculated by Stefan-Boltzmann equation.

$$\phi = \varepsilon A\sigma(T_1^4 - T_2^4) \qquad (1)$$

where ϕ denotes the heat flux, ε denotes emissivity of a dyed towel (0.81), A is the evaporation surface area, σ is the Stefan-Boltzmann constant (5.67×10^{-8} W m^{-2} K^{-4}), T_1 is the temperature of the absorber under 1 kW m^{-2} solar illumination (35 ^{o}C), and T_2 is the ambient temperature (24 ^{o}C). The calculated radiation loss is ~5.7 %.

(b) Convection loss

The convection loss was calculated according to equation 2.

$$Q = hA\Delta T \qquad (2)$$

where Q represents the heat energy, h is the convection heat transfer coefficient (5 W m^{-2} K^{-1}), A is evaporation surface area, ΔT is the temperature difference (11 ^{o}C) between the three-dimensional dyed towel surface and the surrounding environment. Therefore, the calculated convection loss is ~5.5 %.

(c) Conduction loss

The conduction loss was calculated using the following equation.

$$Q = Cm\Delta T \qquad (3)$$

where Q denotes the heat energy, C denotes the specific heat capacity of water (4.2 J g^{-1} $^{o}C^{-1}$), m denotes the water weight and Δt is the temperature change of the bulk water after 45 min evaporation (0.6 ^{o}C). Therefore, the calculated conduction heat loss is ~3.0 %.
